# Methodological quality of cardiac CT and MRI radiomics studies assessed using METRICS and RQS by human readers and ChatGPT 5.1 Thinking

**DOI:** 10.1186/s41747-026-00756-5

**Published:** 2026-06-19

**Authors:** Ludwig Federico Garello, Valentina Giannini, Marco Gatti, Arianna Defeudis, Debora Cafaro, Giulia Nicoletti, Noemi Cristina Culasso, Riccardo Faletti, Andrea Veltri, Renato Cuocolo, Maurizio Balbi

**Affiliations:** 1 Radiology Unit, San Luigi Gonzaga Hospital, Turin, Italy; 2 Department of Oncology, University of Turin, Turin, Italy; 3 Department of Surgical Sciences, University of Turin, Turin, Italy; 4 Azienda Ospedaliero-Universitaria Città della Salute e della Scienza di Torino, Turin, Italy; 5 Candiolo Cancer Institute, FPO-IRCCS, Turin, Italy; 6 Department of Medicine, Surgery and Dentistry, University of Salerno, Baronissi, Italy

**Keywords:** Artificial intelligence, Heart, Magnetic resonance imaging, Radiomics, Tomography (x-ray computed)

## Abstract

**Objective:**

To assess the methodological quality of cardiac CT and MRI radiomics studies using the METhodological RadiomICs Score (METRICS) and Radiomics Quality Score (RQS), and to evaluate inter-rater reliability (IRR) of both scoring tools among human readers and ChatGPT 5.1 Thinking.

**Materials and methods:**

Cardiac CT and MRI radiomics studies published up to 28 February 2025 were scored by human readers with complementary expertise in cardiac imaging and radiomics using both scoring systems. IRR was evaluated in 30 randomly selected studies by two independent groups of secondary readers and ChatGPT 5.1 Thinking.

**Results:**

Of 781 screened records, 154 were included. The overall median METRICS was 0.60 (IQR, 0.52–0.68), and the median percentage RQS was 0.36 (IQR, 0.19–0.42), corresponding to a median absolute RQS of 13 (IQR, 8–15). The scoring systems highlighted several methodological limitations, such as a lack of external validation, a prospective study design, and open data availability. Between human readers, IRR was good for METRICS (ICC, 0.77–0.88) and moderate to good for RQS (ICC, 0.59–0.82). Between human readers and ChatGPT 5.1 Thinking, IRR was moderate to good for METRICS (ICC, 0.70–0.85) but only poor to moderate for RQS (ICC, 0.46–0.56).

**Conclusions:**

Cardiac CT and MRI radiomics research quality was rated as good by METRICS, whereas RQS yielded lower scores. Human readers showed good reproducibility with METRICS and moderate to good reproducibility with RQS. ChatGPT 5.1 Thinking showed potential for automating the evaluation process, but its use requires caution due to potential discrepancies with human evaluations.

**Relevance statement:**

Research quality in cardiac CT and MRI still suffers from substantial limitations. The application of METRICS and RQS using LLMs requires caution, given the limited reproducibility when compared with human assessments.

**Key Points:**

- According to METRICS and RQS, radiomic-based cardiac CT and MRI studies remain affected by substantial methodological limitations.
- Human readers achieved good reproducibility with METRICS and moderate to good reproducibility with RQS.
- ChatGPT 5.1 Thinking may be helpful for scoring radiomics research quality, but its results should be interpreted with caution due to potential discrepancies with human evaluations.

**Graphical Abstract:**

**Figure:**
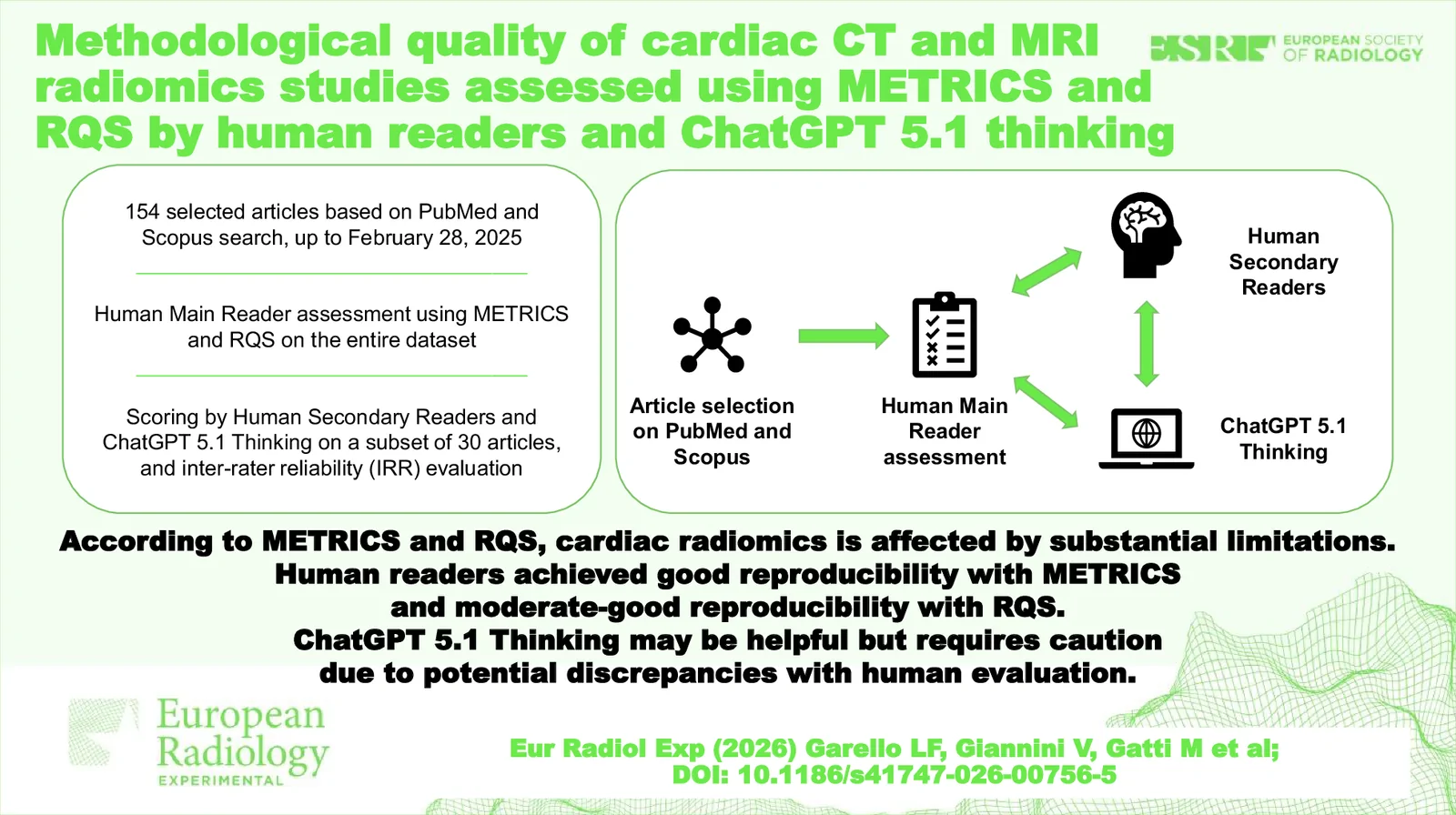

## Background

Radiomics refers to the extraction of high-dimensional quantitative features from medical images to develop prediction models and support clinical decision-making [1]. Over the past decade, this approach has been increasingly applied across several domains, including cardiac computed tomography (CT) and magnetic resonance imaging (MRI) [2–4]. The application of radiomics in these fields holds promise for a substantial clinical impact, as cardiac diseases remain one of the leading causes of morbidity and mortality worldwide [5–7]. Nonetheless, despite a growing body of promising results, no radiomics model applied to cardiac diseases has yet been translated into routine clinical practice. A recognized determinant of this gap is suboptimal research methodology at one or more stages of the radiomic workflow, which may compromise the reliability and reproducibility of published findings [3, 5, 8, 9]. These concerns are particularly relevant because radiomics models involve multiple steps, including preprocessing, segmentation, feature extraction, robustness assessment, and feature selection, each of which may affect the reliability and generalizability of the resulting imaging biomarkers [10].

To promote the quality of radiomics research, various guidelines, checklists, and scoring systems have been proposed [11–13]. The Radiomics Quality Score (RQS), introduced by Lambin et al in 2017, comprises 16 assessment criteria that cover key steps of the radiomics workflow [11]. This tool has been widely used as the primary reference in several systematic reviews evaluating the methodological quality of radiomics studies [14–17]. In 2024, the METhodological RadiomICs Score (METRICS) was introduced by the European Society of Medical Imaging Informatics (EuSoMII) as an assessment tool designed to accommodate a broad range of methodological scenarios in radiomic research, from traditional handcrafted approaches to advanced deep learning models [13]. It includes 30 items divided into nine categories and is available as an online automated calculation tool.

A limited number of studies have examined the quality of cardiac radiomics research [2–4], and, to the best of our knowledge, none have applied both METRICS and RQS to address this issue. Given that these scoring systems emphasize different aspects of the radiomic workflow [18], applying them both may enable a more complete assessment of methodological quality than a single score and help identify potential areas for improvement. Furthermore, there is no evidence regarding the reproducibility of METRICS and RQS in the setting of cardiac radiomics. Growing efforts are being directed toward improving the consistency of these methodological scores, including the use of large language models, such as ChatGPT. However, their integration into radiomics quality assessment is still in its early stages [19–21].

This study aims to: (1) evaluate METRICS and RQS in published cardiac CT and MRI radiomics studies; (2) assess interobserver variability of both scoring systems; (3) explore the performance of ChatGPT 5.1 Thinking in applying RQS and METRICS and assess its agreement with human evaluations.

## Methods

This research was conducted in accordance with the Preferred Reporting Items for Systematic Reviews and Meta-Analyses‒PRISMA checklist. The Institutional Review Board approval was not required because the study involved no patients.

### Search strategy

Three investigators (L.F.G., V.G., and D.C.) conducted a systematic search of the PubMed and Scopus databases to identify articles published up to 28 February 2025. The following search query was used in both databases: “cardiac” AND “radiomics.” A *post hoc* sensitivity search using broader terminology was also performed (the full search strategy and corresponding yield are provided in the Supplementary Material). Duplicates, studies that did not apply either cardiac CT or MRI, non-original studies, articles not published in English, technical papers without a clinical focus, and studies that performed radiomic analyses without developing a radiomic model (*e.g*., those limited to descriptive evaluation of radiomic features) were excluded.

### Human readers scoring and data collection

The METRICS and RQS were applied to all the finally selected studies. Scoring was performed by two readers (L.F.G. and G.N.) with complementary expertise: L.F.G. had 4 years of experience in cardiac imaging, whereas G.N. had 4 years of experience in radiomics applied to medical imaging. Each reader independently scored only the items corresponding to their domain of expertise, as detailed in the Supplementary Material. For each study, the item-level scores from the readers were merged into a single reference score, hereafter referred to as the Main Reader score. The same readers recorded the imaging modality, clinical aim, year of publication, journal quartile, number of patients, and SCImago Journal Rank (SJR) category.

For inter-rater reliability (IRR) analyses, two additional pairs of expert readers (M.B. and A.D., hereafter Secondary Reader A; M.G. and V.G., hereafter Secondary Reader B) evaluated the same randomly selected subgroup of 30 articles [22] using both scoring methods. As for the Main Reader assessment, each secondary reader scored only the items within their domain of expertise, independently and without consensus meetings, and item-level scores within each pair were combined into a single Secondary Reader A and Secondary Reader B score for each study. Secondary readers’ expertise and allocation of items are detailed in the Supplementary Material.

### ChatGPT 5.1 Thinking scoring

ChatGPT 5.1 Thinking was accessed on 8 December 2025 in Turin, Italy, to assess the 30 articles selected for human IRR analyses. Each selected article was uploaded to the ChatGPT web platform in its original PDF or DOC format for analysis, without any further preprocessing. Standardized structured prompts derived from previous studies [19, 21] and designed to cover all METRICS and RQS items were applied to all the selected papers. Each assessment was performed as an independent evaluation to minimize bias using a temporary ChatGPT session with memory disabled. To prevent carry-over effects across evaluations, the session was restarted after completing each prompt. ChatGPT was used with default generation settings, as sampling parameters (*e.g*., temperature/randomness) are not user-configurable in the web application. The full set of prompts is provided in the Supplementary Material.

### Statistical analysis

Differences in METRICS, percentage RQS, and absolute RQS across imaging modality, clinical aim, publication year, journal quartile, number of patients, and SJR category were assessed using the Kruskal–Wallis test on the scores provided by the Main Reader for the entire dataset. The Kruskal–Wallis test was applied when more than two groups with at least ten studies were available; when only two such groups were present, the Mann–Whitney *U* test was applied. In cases of significant overall differences, pairwise Mann–Whitney *U* tests were conducted to evaluate group-wise comparisons. Pairwise analyses were restricted to groups with at least 10 studies.

Differences in METRICS, percentage RQS, and absolute RQS scores among the Main Reader, Secondary Readers, and ChatGPT 5.1 Thinking were assessed using the Friedman test. When significant, *post hoc* pairwise comparisons were performed using Dunn tests, adjusted for multiple comparisons. To assess agreement among readers, we calculated the intraclass correlation coefficient (ICC) using a two-way random effects, absolute agreement, single rater model [22]. The analysis was performed for each reader pair, with corresponding 95% confidence intervals. A *p*-value < 0.05 was considered statistically significant. Statistical analyses were performed in Python 3.8 using the *Pandas* and *NumPy* libraries for data handling, *scipy.stats* and *Pingouin* for statistical testing, *scikit-posthocs* for *post hoc* comparisons, and s*tatsmodels* for IRR assessment (Fleiss’ κ).

## Results

**Fig. 1:**
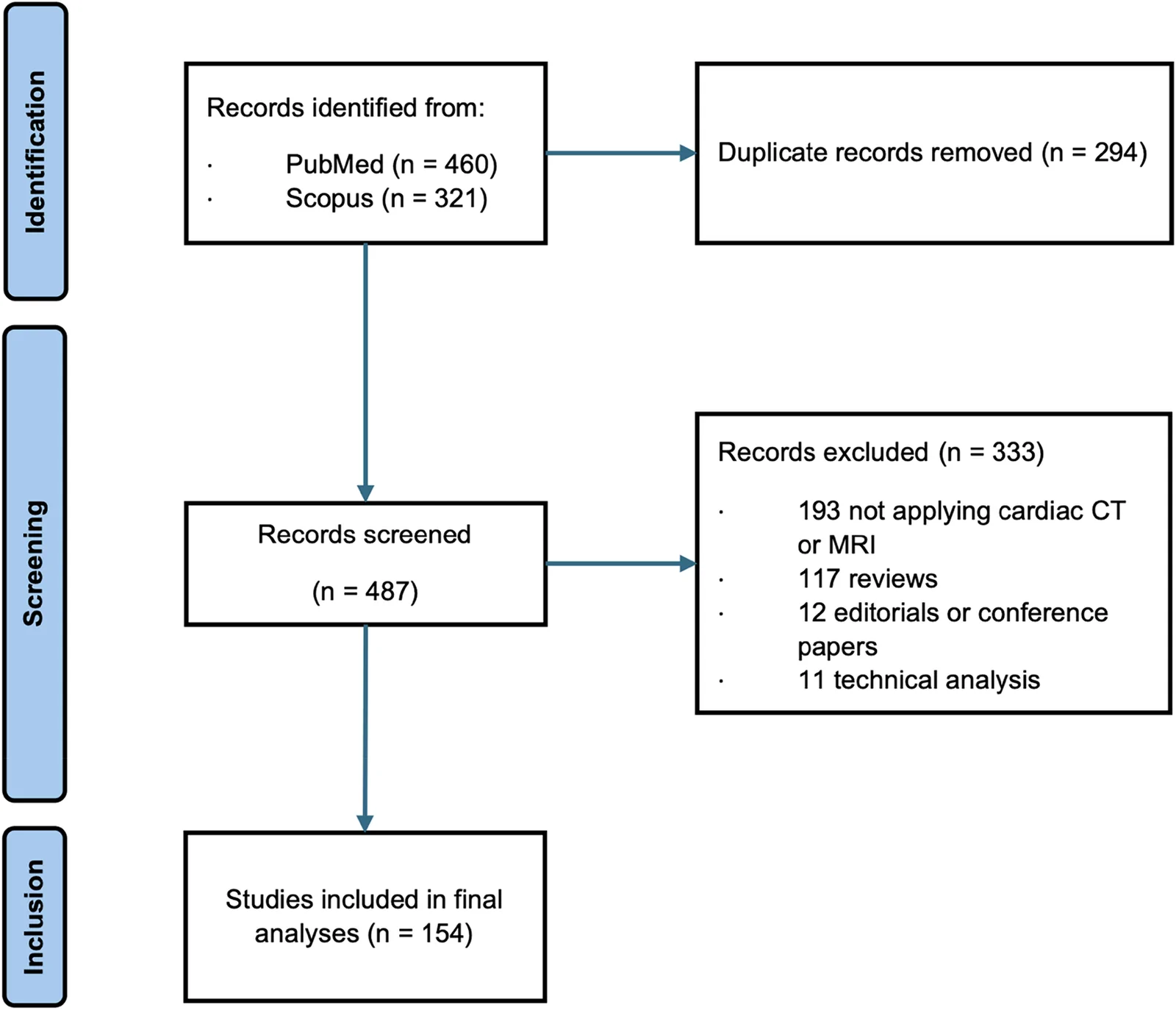
Flow diagram of the literature search and selection process. CT, Computed tomography; MRI, Magnetic resonance imaging

Of 781 articles initially retrieved (460 from PubMed; 321 from Scopus), 154 were selected for final analyses following inclusion and exclusion criteria (Fig. 1).

### Study characteristics

**Fig. 2:**
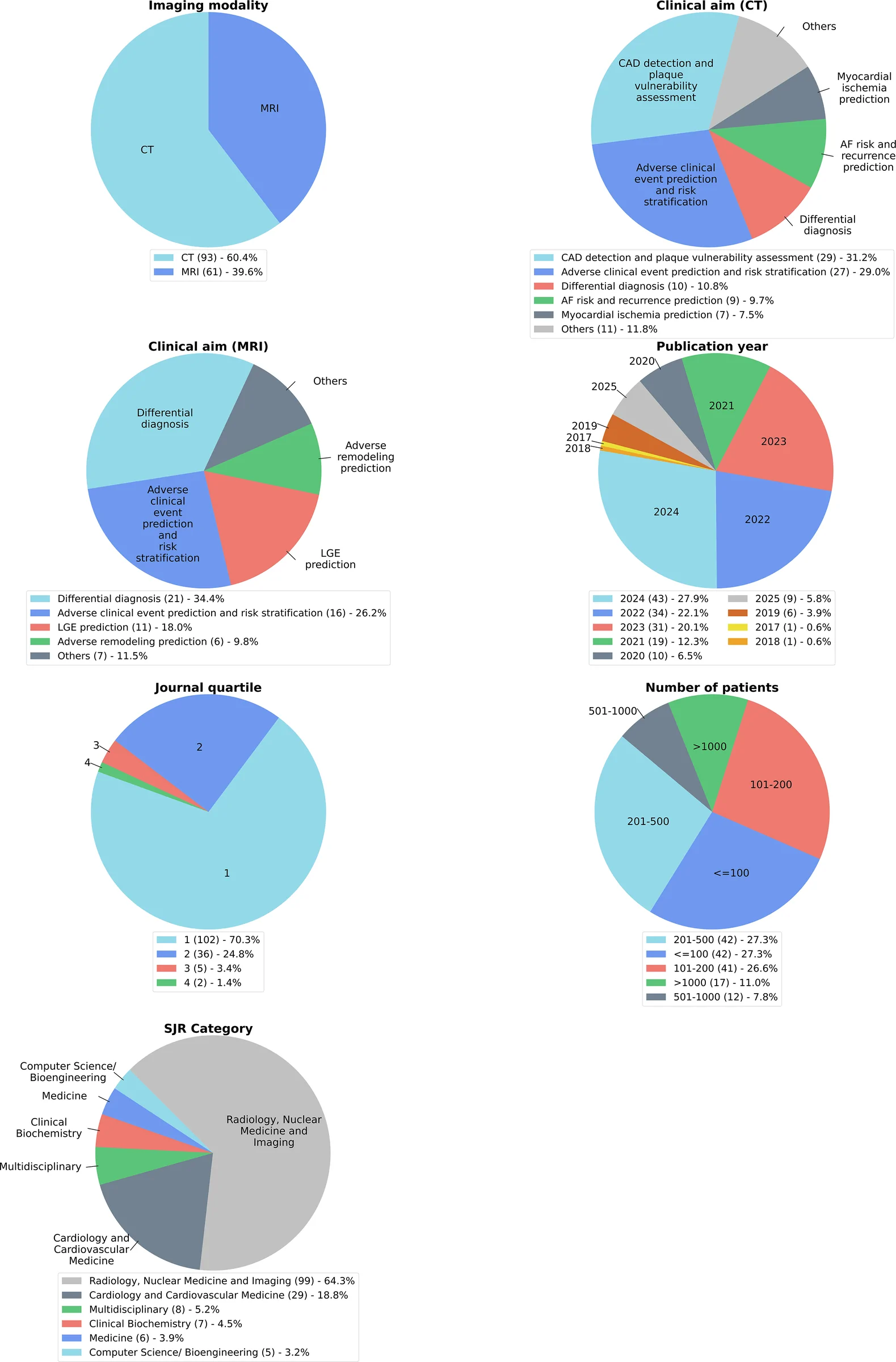
Distribution of studies based on imaging modality, clinical aim, publication year, journal quartile, number of patients, and SJR category. The journal quartile for the year of publication was available at the time of data collection for 145 of 154 studies (94.2%). AF, Atrial fibrillation; CAD, Coronary artery disease; CT, Computed tomography; LGE, Late gadolinium enhancement; MRI, Magnetic resonance imaging; SJR, SCImago Journal Rank

The distribution of included studies by imaging modality, clinical aim, year of publication, journal quartile, number of patients, and SJR category is illustrated in Fig. 2.

CT was the most frequently employed imaging modality, used in 93/154 (60.4%) studies, including 4/154 (2.6%) that used photon-counting CT. Among CT studies, the most common clinical aims were coronary artery disease detection and plaque vulnerability assessment (29/93, 31.2%) and adverse clinical event prediction and risk stratification (27/93, 29.0%), whereas MRI studies most commonly addressed differential diagnosis (21/61, 34.4%) and adverse clinical event prediction and risk stratification (16/61, 26.2%). The majority of studies (108/154, 70.1%) were published between 2022 and 2024. Among the 145 studies for which the journal quartile corresponding to the year of publication was available at the time of data collection, most were published in Q1 (102/145, 70.3%) or Q2 (36/145, 24.8%) journals. Sample sizes were ≤ 100, 101–200, 201–500, 501–1,000, and > 1,000 in, respectively, 42/154 (27.3%), 41/154 (26.6%), 42/154 (27.3%), 12/154 (7.8%), and 17/154 (11.0%) studies. The main SJR category was Radiology, Nuclear Medicine and Imaging (99/154, 64.3%), followed by Cardiology and Cardiovascular Medicine (29/154, 18.8%).

### Study evaluation

According to the Main Reader, the median METRICS was 0.60 (IQR, 0.52–0.68), while the median RQS percentage was 0.36 (IQR, 0.19–0.42), corresponding to a median absolute RQS of 13 (IQR, 8–15).

**Fig. 3:**
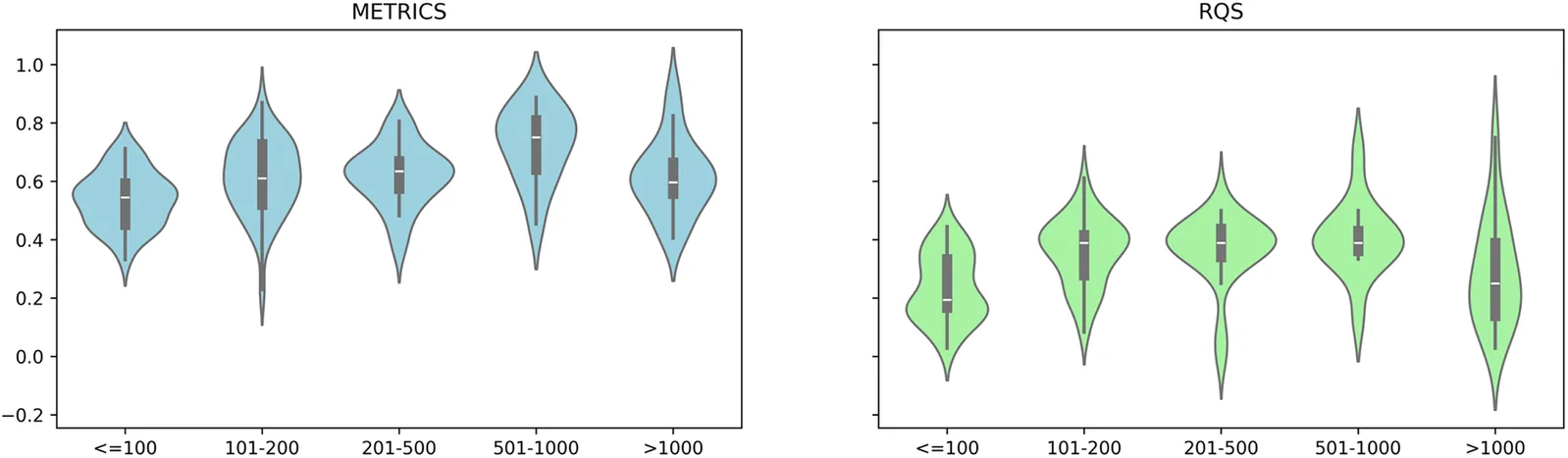
Violin plots showing the distribution of METRICS (left) and RQS (right) scores across studies stratified by the number of patients included. METRICS, METhodological RadiomICs Score; RQS, Radiomics Quality Score

There were no significant differences in METRICS or RQS across imaging modality, year of publication, journal quartile, and SJR category (Table 1). This was also true for clinical aims within each imaging modality (Table 2). The median METRICS of studies with ≤ 100 patients (0.55) was significantly lower than that of studies with larger cohorts (*e.g*., 0.61 for 101–200 patients; *p* = 0.002). Studies with 501–1,000 patients achieved the highest median METRICS (0.75), which was significantly higher than in all other sample sizes (*e.g*., 0.60 for > 1,000 patients, *p* = 0.028). For RQS, the median score of studies with ≤ 100 patients (0.19) was significantly lower than that of larger cohorts (*e.g*., 0.39 for 501–1,000 patients, *p* < 0.001), except for studies including > 1,000 patients (0.25, *p* = 0.387). Violin plots of METRICS and RQS by sample size are shown in Fig. 3.

**Table 1:** Median METRICS, RQS percentage, and RQS points assessed by the Main Reader on the entire dataset, stratified by imaging modality, publication year, journal quartile, number of patients, and SJR category

Imaging modality	METRICS (Median [Q1–Q3])	RQS (Median [Q1–Q3])	RQS points (Median [Q1–Q3])
MRI	0.59 [0.52–0.66]	0.31 [0.19–0.42]	11.00 [7.00–15.00]
CT	0.60 [0.52–0.71]	0.36 [0.24–0.42]	13.00 [8.00–15.00]
*p*-value	0.635	0.204	0.204

Publication year	METRICS (Median [Q1–Q3])	RQS (Median [Q1–Q3])	RQS points (Median [Q1–Q3])
2017	0.57 [0.57–0.57]	0.17 [0.17–0.17]	6.00 [6.00–6.00]
2018	0.44 [0.44–0.44]	0.17 [0.17–0.17]	6.00 [6.00–6.00]
2019	0.60 [0.51–0.67]	0.15 [0.05–0.34]	5.50 [1.75–12.25]
2020	0.65 [0.56–0.74]	0.38 [0.32–0.42]	13.50 [11.50–15.00]
2021	0.56 [0.47–0.68]	0.25 [0.17–0.38]	9.00 [6.00–13.50]
2022	0.59 [0.56–0.65]	0.33 [0.28–0.42]	12.00 [10.00–15.00]
2023	0.65 [0.55–0.69]	0.36 [0.24–0.42]	13.00 [8.50–15.00]
2024	0.60 [0.50–0.69]	0.36 [0.26–0.42]	13.00 [9.50–15.00]
2025	0.64 [0.52–0.65]	0.39 [0.25–0.42]	14.00 [9.00–15.00]
*p*-value	0.257	0.341	0.341

Journal quartile	METRICS (Median [Q1–Q3])	RQS (Median [Q1–Q3])	RQS points (Median [Q1–Q3])
1*	0.61 [0.54–0.69]	0.36 [0.22–0.42]	13.00 [8.00–15.00]
2*	0.59 [0.49–0.68]	0.33 [0.22–0.39]	12.00 [7.75–14.00]
3	0.54 [0.53–0.58]	0.14 [0.11–0.39]	5.00 [4.00–14.00]
4	0.52 [0.48–0.56]	0.25 [0.21–0.29]	9.00 [7.50–10.50]
*p*-value*	0.654	0.327	0.327

Number of patients	METRICS (Median [Q1–Q3])	RQS (Median [Q1–Q3])	RQS points (Median [Q1–Q3])
≤ 100	0.55 [0.45–0.59]	0.19 [0.17–0.33]	7.00 [6.00–12.00]
101–200	0.61 [0.54–0.73]	0.39 [0.28–0.42]	14.00 [10.00–15.00]
201–500	0.64 [0.57–0.67]	0.39 [0.33–0.42]	14.00 [12.00–15.00]
501–1000	0.75 [0.64–0.81]	0.39 [0.36–0.43]	14.00 [13.00–15.50]
> 1000	0.60 [0.56–0.66]	0.25 [0.14–0.39]	9.00 [5.00–14.00]
*p*-value	< 0.001	< 0.001	< 0.001

SJR category	METRICS (Median [Q1–Q3])	RQS (Median [Q1–Q3])	RQS points (Median [Q1–Q3])
Cardiology and cardiovascular medicine*	0.61 [0.52–0.67]	0.36 [0.25–0.42]	13.00 [9.00–15.00]
Clinical biochemistry	0.59 [0.55–0.64]	0.33 [0.32–0.36]	12.00 [11.50–13.00]
Computer science/bioengineering	0.54 [0.51–0.56]	0.17 [0.14–0.28]	6.00 [5.00–10.00]
Medicine	0.50 [0.45–0.70]	0.28 [0.17–0.38]	10.00 [6.25–13.75]
Multidisciplinary	0.57 [0.54–0.70]	0.25 [0.15–0.38]	9.00 [5.25–13.75]
Radiology, nuclear medicine and imaging*	0.60 [0.54–0.68]	0.36 [0.22–0.42]	13.00 [8.00–15.00]
*p*-value*	0.970	0.361	0.361

The *p*-values were calculated using the Kruskal–Wallis or Mann–Whitney tests, as appropriate

“*” indicates *p*-values obtained with the Mann–Whitney test, along with the groups used for comparison

*METRICS* METhodological RadiomICs Score, *RQS* Radiomics Quality Score, *SJR* SCImago Journal Rank

**Table 2:** Median METRICS, RQS percentage, and RQS points assessed by the Main Reader on the entire dataset, stratified by clinical aims within each imaging modality (CT and MRI)

	*n* (%)	METRICS (Median [Q1–Q3])	RQS (Median [Q1–Q3])	RQS Points (Median [Q1–Q3])
**CT**	93/154 (60.4%)	0.60 [0.52–0.71]	0.36 [0.24–0.42]	13.00 [8.00–15.00]
Differential diagnosis	10/93 (10.8%)	0.63 [0.53–0.72]	0.39 [0.22–0.39]	14.00 [8.00–14.00]
Adverse clinical event prediction and risk stratification	27/93 (29.0%)	0.60 [0.56–0.70]	0.36 [0.31–0.40]	13.00 [11.00–14.50]
Coronary artery disease (CAD) detection and plaque vulnerability assessment	29/93 (31.2%)	0.60 [0.53–0.67]	0.36 [0.22–0.42]	13.00 [8.00–15.25]
Myocardial ischemia prediction	7/93 (7.5%)	0.58 [0.55–0.65]	0.36 [0.32–0.42]	13.00 [11.50–15.00]
Atrial fibrillation (AF) risk and recurrence prediction	9/93 (9.7%)	0.60 [0.45–0.71]	0.36 [0.17–0.42]	13.00 [6.00–15.00]
Others	11/93 (11.8%)	0.61 [0.52–0.70]	0.33 [0.28–0.42]	12.00 [10.00–15.00]
*p*-value		0.927	0.987	0.995
**MRI**	61/154 (39.6%)	0.59 [0.52–0.66]	0.31 [0.19–0.42]	11.00 [7.00–15.00]
Differential diagnosis	21/61 (34.4%)	0.61 [0.50–0.67]	0.33 [0.17–0.39]	12.00 [6.00–14.00]
Adverse clinical event prediction and risk stratification	16/61 (26.2%)	0.63 [0.57–0.65]	0.39 [0.24–0.45]	14.00 [8.75–16.25]
Late gadolinium enhancement (LGE) prediction	11/61 (18.0%)	0.59 [0.58–0.73]	0.39 [0.25–0.44]	14.00 [9.00–16.00]
Adverse remodeling prediction	6/61 (9.8%)	0.57 [0.54–0.64]	0.26 [0.25–0.38]	9.50 [9.00–13.75]
Others	7/61 (11.5%)	0.56 [0.47–0.58]	0.11 [0.08–0.25]	4.00 [3.00–9.00]
*p*-value		0.603	0.175	0.175

The *p*-values were calculated using the Kruskal–Wallis test. Comparisons were performed across different aims within the same imaging modality

*CT* Computed tomography*, METRICS* METhodological RadiomICs Score, *MRI* Magnetic resonance imaging, *RQS* Radiomics Quality Score

**Fig. 4:**
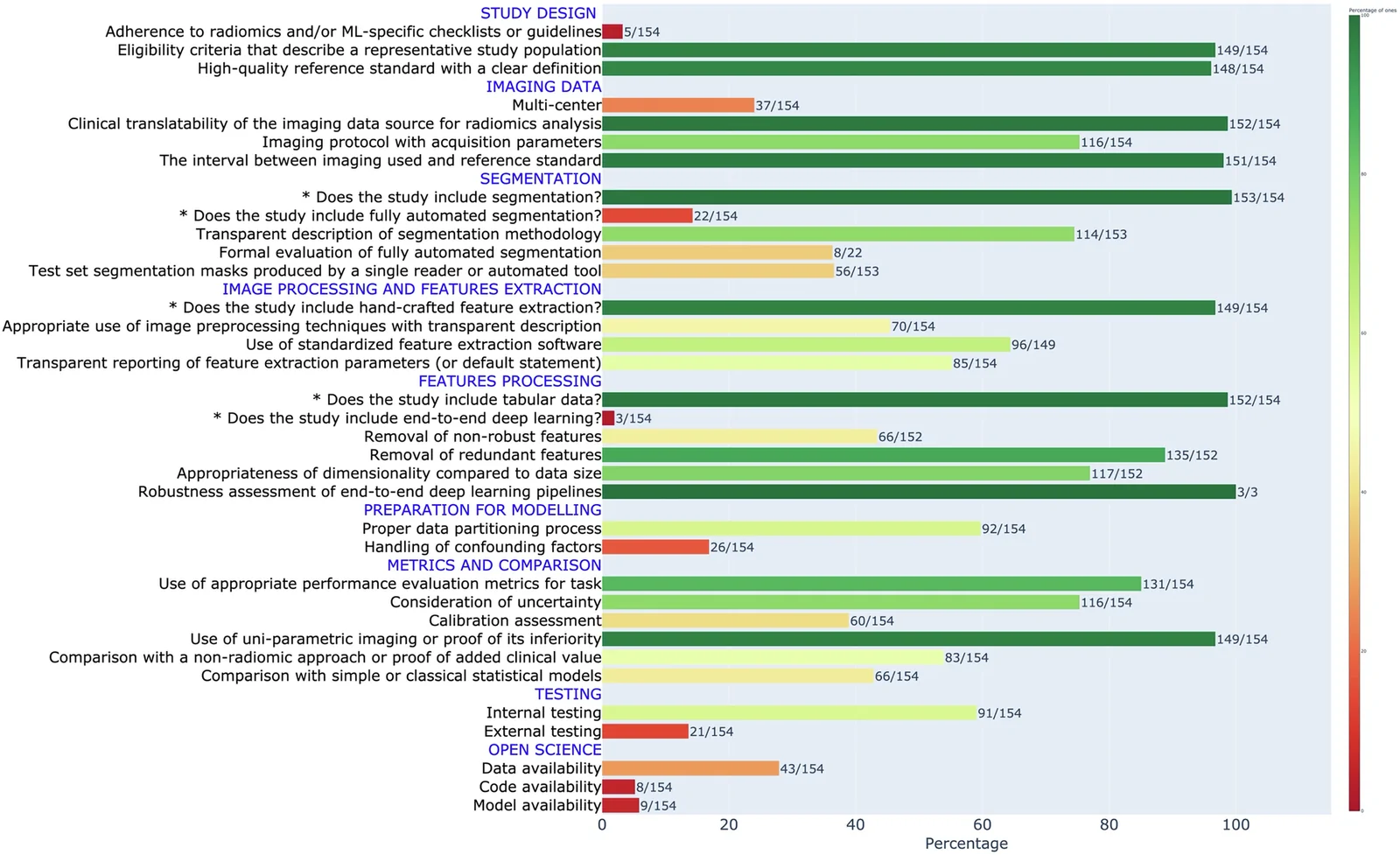
Distribution of METRICS item compliance according to the Main Reader. Bars indicate the proportion of studies satisfying each methodological criterion, grouped by domain. “*” indicates conditions. METRICS, METhodological RadiomICs Score

Figure 4 illustrates the percentage of papers satisfying each METRICS item according to the Main Reader. The median number of applicable items per study was 28 (IQR, 28–28). Only 5/154 (3.2%) of studies adhered to specific radiomic guidelines or checklists. Most studies clearly defined eligibility criteria describing a representative study population (149/154, 96.7%) and provided a high-quality reference standard (148/154, 96.1%). Nearly all studies (152/154, 98.7%) used data sources considered clinically translatable for radiomics analysis. Fully automated segmentation was uncommon (22/154, 14.3%), with only 8/22 (36.4%) studies reporting a formal evaluation of the segmentation masks. Among 153 studies that segmented regions of interest, 114 (74.5%) provided a transparent description of the segmentation methodology. Transparency in reporting image preprocessing steps and feature-extraction parameters was limited (85/154, 55.2%). Regarding feature processing, only 3/154 (1.9%) studies included end-to-end deep learning. Most removed redundant features (135/152, 88.8%), whereas removal of non-robust features was less frequent (66/152, 43.4%). Of 152 studies that removed either non-robust or redundant features, 117 (77%) reached an appropriate dimensionality of features. For testing, external validation was performed in only 21/154 (13.6%) studies, while internal testing was used in 91/154 (59.1%) studies. Code, model, and data were openly available in 8/154 (5.2%), 9/154 (5.8%), and 43/154 (27.9%) studies, respectively.

**Fig. 5:**
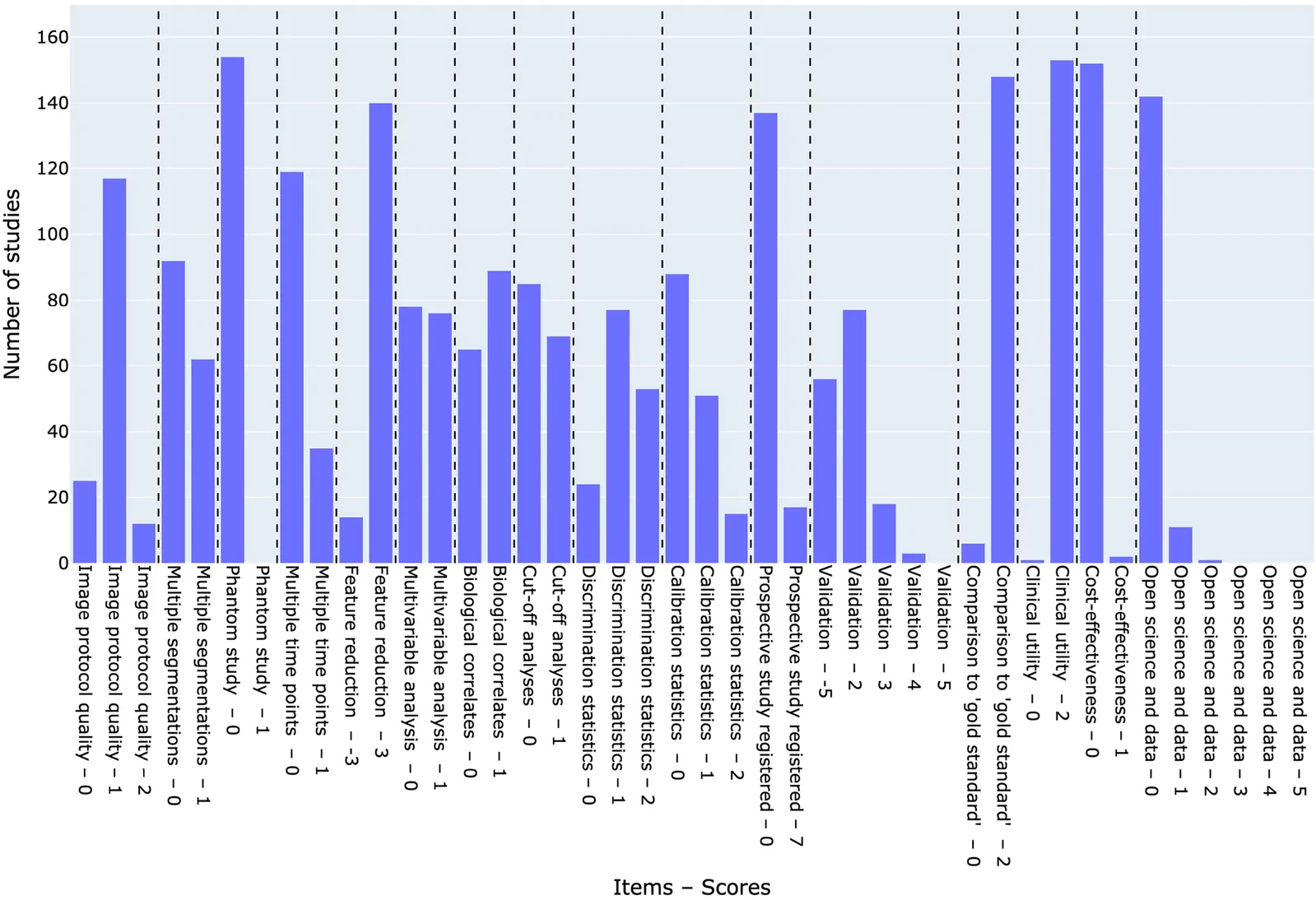
Distribution of the number of studies across the different RQS items and score levels, according to the Main Reader. RQS, Radiomics Quality Score

Figure 5 shows the distribution of RQS points across the different evaluation criteria according to the Main Reader. No studies employed phantoms. Feature reduction was applied in most cases (140/154, 90.9%), whereas a prospective design was uncommon (17/154, 11.0%). Regarding validation, 56/154 (36.4%) studies did not report any strategy, 77/154 (50.0%) performed only internal validation using data from one center, 18/154 (11.7%) used data from a different institution, and only 3/154 (1.9%) relied on two independent external datasets. Comparison with a reference standard was performed in 148/154 (96.1%) studies, and almost all reports (153/154, 99.3%) discussed model applicability in a clinical setting. Cost-effectiveness analysis was reported in only 2/154 (1.3%) studies. Open data and model sharing were largely lacking, with 142/154 (92.2%) studies failing to provide any of the requested elements (scans, segmentations, codes, or radiomic features).

### Scoring agreement

Median METRICS and RQS showed no statistically significant differences across human readers (*p* = 0.821 and *p* = 0.808, respectively). ChatGPT 5.1 Thinking-generated median METRICS was not significantly different than those of human readers (*p* = 0.918). ChatGPT 5.1 Thinking yielded lower median percentage RQS values than all human reader groups, with statistically significant differences compared with the Main Reader and Secondary Reader A (*p* = 0.033 and *p* = 0.038, respectively), but not with Secondary Reader B (*p* = 0.115) (Table 3). Similarly, ChatGPT 5.1 Thinking-generated median absolute RQS was significantly lower than that of the Main Reader (*p* = 0.026) and Secondary Reader A (*p* = 0.019), whereas the difference compared with Secondary Reader B did not reach statistical significance (*p* = 0.094) (Table 3).

**Table 3:** Median METRICS, RQS percentage, and RQS points assigned by human readers (Main Reader and Secondary Readers A and B) and GPT 5.1 Thinking on a randomly selected subset of 30 studies

	METRICS (Median [Q1–Q3])	RQS (Median [Q1–Q3])	RQS Points (Median [Q1–Q3])
Main Reader	0.64 [0.52–0.73]	0.39 [0.25–0.42]	14.00 [9.00–15.00]
Secondary Reader A	0.65 [0.56–0.72]	0.39 [0.22–0.42]	14.00 [8.00–15.00]
Secondary Reader B	0.61 [0.54–0.72]	0.39 [0.20–0.41]	14.00 [7.25–14.75]
ChatGPT 5.1 Thinking	0.62 [0.50–0.75]	0.27 [0.12–0.33]	9.50 [4.25–12.00]

*METRICS* METhodological RadiomICs Score, *RQS* Radiomics Quality Score

ICCs between human readers and ChatGPT 5.1 Thinking using both scoring tools are reported in Table 4. For METRICS, IRR was good between Main Reader and Secondary Readers (ICC 0.77 [95% CI: 0.56, 0.89] with Secondary Reader A; ICC 0.88 [95% CI: 0.76, 0.94] with Secondary Reader B) and good between the Secondary Readers (ICC 0.88 [95% CI: 0.75, 0.94]). ChatGPT 5.1 showed good IRR with both Main Reader and Secondary Reader B (ICC 0.85 [95% CI: 0.70, 0.92]; ICC 0.81 [95% CI: 0.63, 0.90]), and moderate IRR with Secondary Reader A (ICC 0.70 [95% CI: 0.46, 0.85]).

**Table 4:** ICC for inter-rater reliability between human readers (Main Reader and Secondary Readers A and B) and ChatGPT 5.1 Thinking using METRICS and RQS on a randomly selected subset of 30 studies

	ICC METRICS [95% CI]	*p*-value	ICC RQS [95% CI]	*p*-value
Main Reader *vs* Secondary Reader A	0.77 [0.56, 0.89]	< 0.001	0.59 [0.30, 0.78]	< 0.001
Main Reader *vs* Secondary Reader B	0.88 [0.76, 0.94]	< 0.001	0.63 [0.36, 0.81]	< 0.001
Secondary Reader A *vs* Secondary Reader B	0.88 [0.75, 0.94]	< 0.001	0.82 [0.66, 0.91]	< 0.001
Main Reader *vs* ChatGPT 5.1 Thinking	0.85 [0.70, 0.92]	< 0.001	0.46 [0.03, 0.72]	< 0.001
Secondary Reader A *vs* ChatGPT 5.1 Thinking	0.70 [0.46, 0.85]	< 0.001	0.56 [0.00, 0.82]	< 0.001
Secondary Reader B *vs* ChatGPT 5.1 Thinking	0.81 [0.63, 0.90]	< 0.001	0.56 [0.09, 0.79]	< 0.001

*ICC* Intraclass correlation coefficient, *METRICS* METhodological RadiomICs Score, *RQS* Radiomics Quality Score

For both percentage and absolute RQS, IRR was moderate between Main Reader and Secondary Readers (ICC 0.59 [95% CI: 0.30, 0.78] with Secondary Reader A; ICC 0.63 [95% CI: 0.36, 0.81] with Secondary Reader B) and good between the Secondary Readers (ICC 0.82 [95% CI: 0.66, 0.91]). ChatGPT 5.1 Thinking showed poor IRR with Main Reader (ICC 0.46 [95% CI: 0.03, 0.72]) and moderate with both Secondary Readers (ICC 0.56 [95% CI: 0.00, 0.82] with Secondary Reader A; ICC 0.56 [95% CI: 0.09, 0.79] with Secondary Reader B).

Figure S1 illustrates the proportion of studies that received positive scores for each METRICS item according to human readers and ChatGPT 5.1 Thinking. Figure S2 depicts the item-level distributions of RQS assessments for the same studies, expressed as study counts by score category.

## Discussion

Radiomics holds promise for overcoming the limitations of subjective assessment in cardiac CT and MRI. To promote its clinical translation through methodological improvement, dedicated scoring systems such as RQS and METRICS have been developed. Nonetheless, to the best of our knowledge, no prior study has applied both nor evaluated their reproducibility in cardiac radiomics. ChatGPT has shown potential to facilitate the large-scale assessment of these scoring systems [19, 20], yet its use in this context remains largely unexplored.

The present study found that the methodological quality of cardiac CT and MRI radiomics research published up to 28 February 2025 was rated as good by METRICS (median score: 0.60 [IQR, 0.52–0.68]), while RQS yielded lower scores (median score: 0.36 [IQR, 0.19–0.42]). Human readers showed good reproducibility for METRICS and moderate to good reproducibility for RQS. ChatGPT 5.1 Thinking assigned similar METRICS scores to those assigned by human readers, whereas RQS scores were lower. Agreement between human readers and ChatGPT 5.1 Thinking was rated as moderate to good using METRICS, but as poor to moderate using RQS.

The median METRICS score observed in our analyses is consistent with a recent report assessing CT and MRI radiomics for cardiovascular risk prediction, in which the average METRICS total score was 54.52 ± 15.89% [23]. In that study, however, the evaluation of research quality was a secondary aim, with no sub-analyses other than assessing associations with publication year and study category. Moreover, no comparison with RQS was reported, and reproducibility analyses were not performed. In keeping with that prior report and other analyses in different domains [23–26], METRICS highlighted several methodological limitations, including limited adherence to checklists or guidelines, lack of formal evaluation of fully automated segmentations, limited external testing, and limited availability of data, code, and models.

Prior meta-research has reported improvements in the methodological quality of radiomics research over time [27, 28], and earlier RQS-based reports on cardiac radiomics suggested similar—albeit not statistically significant—trends toward improvement [2–4]. Nevertheless, our findings do not provide clear evidence of improved adherence to good methodological practice in cardiac radiomics over time, in line with recent evidence [23]. The true impact of METRICS on the quality of radiomics research remains to be determined, given its recent introduction. Future studies are expected to clarify this point and to explore additional strategies, such as interpretative guidance on scoring tools [29] or structured checklists [30], to improve methodological standards in the field.

The RQS scores reported in the present review were slightly higher than those reported in previous analyses of cardiac radiomics research, yet remained below desirable levels. In a study evaluating 32 cardiac MRI articles, the mean overall RQS was 5.16 ± 5.85 (percentage score 14.3 ± 16.3%) [4]. Lee et al reported a mean RQS of 9.9 ± 7.3 (percentage score 27.4%) across 15 cardiac CT studies [2]. More recently, Ponsiglione et al assigned a median RQS of 7 (IQR, 4–12; percentage score 19.4%) to 53 articles on either cardiac CT or MRI [3]. Consistent with these findings, we found that most articles provided comprehensive details of the imaging protocols and applied feature reduction to decrease the risk of overfitting. Conversely, feature robustness to scanner or temporal variability was tested in a few cases, and no studies employed phantoms, which accounts for the predominant retrospective nature of the included studies. Nonetheless, evaluating feature robustness with respect to scan parameter variability and other confounders, such as organ motion, may be challenging even when data are prospectively collected [31–33]. Considering that these tasks are most often performed as standalone technical studies rather than embedded in clinically oriented radiomic research, the exclusion of technical papers may have affected RQS scoring in our review. Other areas for improvement included cost-effectiveness analyses and the availability of open data, both of which, in line with prior reports [3, 4], were lacking in nearly all the selected articles.

METRICS yielded a higher overall methodological quality than RQS. Similar trends have been reported in other domains of radiomic research. For instance, Russo et al evaluated radiomics studies on endometrial cancer and reported an average RQS of 30.5% compared with a METRICS of 67.6% [34]. In an analysis of radiomics-based studies on bone chondrosarcoma, METRICS and RQS adherence rates ranged from 37.3% to 94.8% and from 2.8% to 44.4%, respectively [35]. These differences may reflect how the two scoring systems allocate weight to individual items: RQS places a significant portion of its weight on a few criteria, whereas METRICS provides a more balanced quality assessment [13, 18]. The gap is further amplified by the non-linear conversion of absolute into percentage scores in RQS, in contrast to the linear scaling adopted by METRICS [18]. The penalization of deep learning studies, in which segmentation-related items are not applicable, may also contribute to lower RQS scores [18]. Nevertheless, since only a few studies used deep learning methods, the influence of this factor on our findings was likely negligible.

Previous studies have reported variable IRR for both METRICS and RQS when these tools were applied by human readers with different levels of expertise. Dedicated reproducibility studies demonstrated poor to moderate IRR for both scoring systems [36, 37], whereas reproducibility assessed within systematic quality audits has yielded more encouraging results [26, 34]. In our study, we observed good agreement for METRICS and moderate to good agreement for RQS between human raters; however, the raters’ high level of expertise may limit the generalizability of the results to less controlled settings.

ChatGPT 5.1 Thinking demonstrated moderate to good agreement with human readers for METRICS, suggesting—consistent with previous studies [19, 21]—its potential role in supporting the automated evaluation of radiomic research quality. By contrast, agreement between ChatGPT 5.1 Thinking and human readers for RQS was only poor to moderate. This finding differs from the results of Mese and Kocak, who reported high agreement between ChatGPT and human raters using RQS [19]. Although beyond the scope of the present article, our analysis suggests that when METRICS items are grounded in subjective judgment rather than quantitative evidence, ChatGPT 5.1 Thinking tends to overestimate human readers’ assessments (*e.g*., 90% *versus* 40–66.7% for *transparent* reporting of feature-extraction parameters; 100% *versus* 66.7–83.3% for *transparent* description of the segmentation methodology). Conversely, ChatGPT 5.1 Thinking tends to underestimate RQS items that require clinical judgment (*e.g*., “biological correlates” was scored as 0 points in 25 studies by ChatGPT 5.1 Thinking *versus* 6–13 by human readers, and “comparison to gold standard” was scored as 0 points in 11 studies by ChatGPT 5.1 Thinking *versus* 1–2 by human readers). To improve the trustworthiness of such tools, it may be beneficial to require that each assigned score be supported by data-driven excerpts from the source articles (*e.g*., quoted methods, key results, or figures), enabling independent verification of the underlying decisions. By improving the interpretability of discrepancies, this approach could support an “augmented radiologist” framework—AI could speed up routine or time-consuming tasks, while clinicians would retain ultimate judgment.

This study had limitations. First, the analyses focused on methodological quality; reporting quality, for instance, using the CheckList for EvaluAtion of Radiomics Research (CLEAR) [12], was not assessed. Second, reproducibility analyses did not include intra-reader analyses and were performed by expert reviewers, with no evaluation of less experienced readers. Third, certain studies were conducted prior to the adoption of the RQS, and most were published before the METRICS and related guidance (*e.g*., METRICS-E3) became available; this timing may have influenced scoring and should be considered when interpreting adherence to these frameworks. Fourth, we did not assess the recently introduced RQS 2.0 [38]; however, this score was not available at the time of analysis. Finally, large language model performance was evaluated only for ChatGPT 5.1 Thinking using a fixed prompt structure, which limits the generalizability of the study findings.

In conclusion, radiomics-based cardiac CT and MRI studies remain affected by substantial methodological limitations. Human readers showed good reproducibility for METRICS and moderate to good reproducibility for RQS. ChatGPT 5.1 Thinking may be helpful for scoring radiomics research quality, but its results should be interpreted with caution due to potential discrepancies with human evaluations.

## Supplementary information

**Additional File 1: Supplementary Figure 1.** Proportion of METRICS items scored as positive by the readers (Main Reader, Secondary Readers A and B, ChatGPT 5.1 Thinking) on 30 randomly selected studies. **Supplementary Figure 2.** Distribution of the number of studies across the different RQS items and score levels, according to the readers (Main Reader, Secondary Readers A and B, ChatGPT 5.1 Thinking) on 30 randomly selected studies.

## Data Availability

The datasets used and/or analyzed during the current study are available from the corresponding author on reasonable request.

## Abbreviations

CT: Computed tomography
ICC: Intraclass correlation coefficient
IRR: Inter-rater reliability
METRICS: METhodological RadiomICs Score
MRI: Magnetic resonance imaging
RQS: Radiomics quality score
SJR: SCImago Journal Rank

## References

[1] Gillies RJ, Kinahan PE, Hricak H (2016) Radiomics: images are more than pictures, they are data. Radiology 278:563–577. 10.1148/radiol.2015151169 PMC4734157 26579733

[2] Lee S, Han K, Suh YJ (2022) Quality assessment of radiomics research in cardiac CT: a systematic review. Eur Radiol 32:3458–3468. 10.1007/s00330-021-08429-0 34981135

[3] Ponsiglione A, Stanzione A, Cuocolo R et al (2022) Cardiac CT and MRI radiomics: systematic review of the literature and radiomics quality score assessment. Eur Radiol 32:2629–2638. 10.1007/s00330-021-08375-x 34812912

[4] Chang S, Han K, Suh YJ, Choi BW (2022) Quality of science and reporting for radiomics in cardiac magnetic resonance imaging studies: a systematic review. Eur Radiol 32:4361–4373. 10.1007/s00330-022-08587-9 35230519

[5] Baeßler B, Engelhardt S, Hekalo A et al (2024) Perfect match: radiomics and artificial intelligence in cardiac imaging. Circ Cardiovasc Imaging 17:e015490. 10.1161/CIRCIMAGING.123.015490 38889216

[6] Timmis A, Aboyans V, Vardas P et al (2024) European Society of Cardiology: the 2023 atlas of cardiovascular disease statistics. Eur Heart J 45:4019–4062. 10.1093/eurheartj/ehae466 39189413

[7] Martin SS, Aday AW, Allen NB et al (2025) 2025 Heart disease and stroke statistics: a report of US and global data from the American Heart Association. Circulation 151:e41–e660. 10.1161/CIR.0000000000001303 PMC12256702 39866113

[8] Cuocolo R, Imbriaco M (2021) Machine learning solutions in radiology: does the emperor have no clothes? Eur Radiol 31:3783–3785. 10.1007/s00330-021-07895-w 33856518

[9] Corti A, Lo Iacono F, Ronchetti F et al (2025) Enhancing cardiovascular risk stratification: radiomics of coronary plaque and perivascular adipose tissue—current insights and future perspectives. Trends Cardiovasc Med 35:47–59. 10.1016/j.tcm.2024.06.003 38960074

[10] Rundo L, Militello C (2024) Image biomarkers and explainable AI: handcrafted features *versus* deep learned features. Eur Radiol Exp 8:130. 10.1186/s41747-024-00529-y PMC11576747 39560820

[11] Lambin P, Leijenaar RTH, Deist TM et al (2017) Radiomics: the bridge between medical imaging and personalized medicine. Nat Rev Clin Oncol 14:749–762. 10.1038/nrclinonc.2017.141 28975929

[12] Kocak B, Baessler B, Bakas S et al (2023) CheckList for EvaluAtion of Radiomics research (CLEAR): a step-by-step reporting guideline for authors and reviewers endorsed by ESR and EuSoMII. Insights Imaging 14:75. 10.1186/s13244-023-01415-8 PMC10160267 37142815

[13] Kocak B, Akinci D’Antonoli T, Mercaldo N et al (2024) METhodological RadiomICs Score (METRICS): a quality scoring tool for radiomics research endorsed by EuSoMII. Insights Imaging 15:8. 10.1186/s13244-023-01572-w PMC10792137 38228979

[14] Spadarella G, Calareso G, Garanzini E et al (2021) MRI based radiomics in nasopharyngeal cancer: systematic review and perspectives using radiomic quality score (RQS) assessment. Eur J Radiol 140:109744. 10.1016/j.ejrad.2021.109744 33962253

[15] Tran K, Ginzburg D, Hong W et al (2024) Post-radiotherapy stage III/IV non-small cell lung cancer radiomics research: a systematic review and comparison of CLEAR and RQS frameworks. Eur Radiol 34:6527–6543. 10.1007/s00330-024-10736-1 PMC11399214 38625613

[16] Staal FCR, Aalbersberg EA, Van Der Velden D et al (2022) GEP-NET radiomics: a systematic review and radiomics quality score assessment. Eur Radiol 32:7278–7294. 10.1007/s00330-022-08996-w 35882634

[17] Yuan E, Chen Y, Song B (2023) Quality of radiomics for predicting microvascular invasion in hepatocellular carcinoma: a systematic review. Eur Radiol 33:3467–3477. 10.1007/s00330-023-09414-5 36749371

[18] Koçak B, D’Antonoli TA, Cuocolo R (2024) Exploring radiomics research quality scoring tools: a comparative analysis of METRICS and RQS. Diagn Interv Radiol 30:366–369. 10.4274/dir.2024.242793 PMC11589524 38700426

[19] Mese I, Kocak B (2024) ChatGPT as an effective tool for quality evaluation of radiomics research. Eur Radiol 35:2030–2042. 10.1007/s00330-024-11122-7 39406959

[20] De Almeida JG, Papanikolaou N (2025) Auto-METRICS: LLM-assisted scientific quality control for radiomics research. Eur J Radiol 191:112358. 10.1016/j.ejrad.2025.112358 40812087

[21] Mese I, Kocak B (2025) Large language models in methodological quality evaluation of radiomics research based on METRICS: ChatGPT *vs* NotebookLM *vs* radiologist. Eur J Radiol 184:111960. 10.1016/j.ejrad.2025.111960 39938163

[22] Koo TK, Li MY (2016) A guideline of selecting and reporting intraclass correlation coefficients for reliability research. J Chiropr Med 15:155–163. 10.1016/j.jcm.2016.02.012 PMC4913118 27330520

[23] Cavallo AU, Ponsiglione A, Pereira B et al (2025) CT and MRI radiomics in cardiovascular risk prediction: a systematic review and meta-analysis by the EuSoMII Radiomics Auditing Group. Eur Radiol. 10.1007/s00330-025-12236-2 PMC13086800 41441995

[24] Deng K, Chen T, Leng Z et al (2024) Radiomics as a tool for prognostic prediction in transarterial chemoembolization for hepatocellular carcinoma: a systematic review and meta-analysis. Radiol Med 129:1099–1117. 10.1007/s11547-024-01840-9 PMC11322429 39060885

[25] Renjifo-Correa ME, Fanni SC, Bustamante-Cristancho LA et al (2025) Diagnostic accuracy of radiomics in the early detection of pancreatic cancer: a systematic review and qualitative assessment using the methodological radiomics score (METRICS). Cancers (Basel) 17:803. 10.3390/cancers17050803 PMC11898638 40075651

[26] Aghakhanyan G, Filidei T, Febi M et al (2024) Advancing pediatric sarcomas through radiomics: a systematic review and prospective assessment using radiomics quality score (RQS) and methodological radiomics score (METRICS). Diagnostics (Basel) 14:832. 10.3390/diagnostics14080832 PMC11049622 38667477

[27] Barry N, Kendrick J, Molin K et al (2025) Evaluating the impact of the radiomics quality score: a systematic review and meta-analysis. Eur Radiol 35:1701–1713. 10.1007/s00330-024-11341-y PMC11835903 39794540

[28] Kocak B, Keles A, Kose F, Sendur A (2024) Quality of radiomics research: comprehensive analysis of 1574 unique publications from 89 reviews. Eur Radiol 35:1980–1992. 10.1007/s00330-024-11057-z 39237770

[29] Kocak B, Ammirabile A, Ambrosini I et al (2025) Explanation and elaboration with examples for METRICS (METRICS-E3): an initiative from the EuSoMII Radiomics Auditing Group. Insights Imaging 16:175. 10.1186/s13244-025-02061-y PMC12351001 40802002

[30] Kocak B, Borgheresi A, Ponsiglione A et al (2024) Explanation and elaboration with examples for CLEAR (CLEAR-E3): an EuSoMII Radiomics Auditing Group Initiative. Eur Radiol Exp 8:72. 10.1186/s41747-024-00471-z PMC11091004 38740707

[31] Alis D, Yergin M, Asmakutlu O et al (2021) The influence of cardiac motion on radiomics features: radiomics features of non-enhanced CMR cine images greatly vary through the cardiac cycle. Eur Radiol 31:2706–2715. 10.1007/s00330-020-07370-y 33051731

[32] Adachi T, Nagasawa R, Nakamura M et al (2022) Vulnerabilities of radiomic features to respiratory motion on four-dimensional computed tomography-based average intensity projection images: a phantom study. J Appl Clin Med Phys 23:e13498. 10.1002/acm2.13498 PMC8906211 35088515

[33] Chen Y, Kan K, Liu S et al (2023) Impact of respiratory motion on^18^F-FDG PET radiomics stability: clinical evaluation with a digital PET scanner. J Appl Clin Med Phys 24:e14200. 10.1002/acm2.14200 PMC10691638 37937706

[34] Russo L, Bottazzi S, Kocak B et al (2024) Evaluating the quality of radiomics-based studies for endometrial cancer using RQS and METRICS tools. Eur Radiol 35:202–214. 10.1007/s00330-024-10947-6 PMC11632020 39014086

[35] Gitto S, Cuocolo R, Klontzas ME et al (2025) Quality appraisal of radiomics-based studies on chondrosarcoma using methodological radiomics score (METRICS) and radiomics quality score (RQS). Insights Imaging 16:129. 10.1186/s13244-025-02016-3 PMC12177113 40533701

[36] Akinci D’Antonoli T, Cavallo AU, Kocak B et al (2025) Reproducibility of methodological radiomics score (METRICS): an intra- and inter-rater reliability study endorsed by EuSoMII. Eur Radiol 35:4533–4545. 10.1007/s00330-025-11443-1 PMC12226626 39969552

[37] Akinci D’Antonoli T, Cavallo AU, Vernuccio F et al (2023) Reproducibility of radiomics quality score: an intra- and inter-rater reliability study. Eur Radiol 34:2791–2804. 10.1007/s00330-023-10217-x PMC10957586 37733025

[38] Lambin P, Woodruff HC, Mali SA et al (2025) Radiomics Quality Score 2.0: towards radiomics readiness levels and clinical translation for personalized medicine. Nat Rev Clin Oncol. 10.1038/s41571-025-01067-1 40903523

