# Supplementary material for: Methodological quality of cardiac CT and MRI radiomics studies assessed using METRICS and RQS by human readers and ChatGPT 5.1 Thinking
Source: Eur Radiol Exp. 2026 Jun 19;10:95. doi: 10.1186/s41747-026-00756-5 (PMC13282432; doi:10.1186/s41747-026-00756-5)
Supplement: Supplementary file 1 — Additional File 1: Supplementary Figure 1. Proportion of METRICS items scored as positive by the readers (Main Reader, Secondary Readers A and B, ChatGPT 5.1 Thinking) on 30 randomly selected studies. Supplementary Figure 2. Distribution of the number of studies across the different RQS items and score levels, according to the readers (Main Reader, Secondary Readers A and B, ChatGPT 5.1 Thinking) on 30 randomly selected studies. [file 41747_2026_756_MOESM1_ESM.pdf]

Methodological quality of cardiac CT and MRI radiomics studies assessed using METRICS and RQS by human readers and ChatGPT 5.1 Thinking

ELECTRONIC SUPPLEMENTARY MATERIAL

Expanded search strategy and post hoc sensitivity search results

The expanded search string was: (machine learning OR radiomics) AND (“cardiac” OR “heart”) AND (“computed tomography” OR “CT” OR “magnetic resonance” OR “MRI” OR “MR”). Using this string, we retrieved n = 2402 records in PubMed and n = 8376 in Scopus. Compared with the primary search strategy, the sensitivity search identified n = 14 potentially eligible records after application of the prespecified exclusion criteria. The results of this post hoc sensitivity search were not incorporated into the main screening workflow; therefore, they were not included in the final study sample and were not analyzed.

Allocation of METRICS and RQS items according to reader expertise

METRICS

G.N., A.D., and V.G. evaluated the following items:

- Conditional items
- Segmentation
- Image Processing and Feature Extraction
- Feature Processing
- Preparation for Modeling
- Metrics and Comparison (Items #20, 21, 22, and 25)
- Testing

L.F.G., M.G., and M.B. evaluated the following items:

- Study Design
- Imaging Data
- Metrics and Comparison (Items #23 and 24)
- Open Science

RQS

G.N., A.D., and V.G. evaluated the following items:

- Multiple segmentations
- Feature reduction or adjustment for multiple testing
- Multivariable analysis with non-radiomics features
- Discrimination statistics
- Calibration statistics
- Validation

L.F.G., M.G., and M.B. evaluated the following items:

- Image protocol quality
- Phantom study on all scanners
- Imaging at multiple time points
- Detect and discuss biological correlates
- Cut-off analyses
- Prospective study registered in a trial database
- Comparison method
- Potential clinical utility
- Cost-effectiveness analysis
- Open science and data

Secondary readers expertise

M.G. has over 7 years of experience in cardiac imaging, M.B. has 5 years of experience in cardiothoracic imaging, and A.D. and V.G. have, respectively, 6 and more than 12 years of experience in radiomics applied to medical imaging.

METRICS prompt

In the present study, we used the same METRICS prompt as Mese I and Kocak B [22]. In their work, the METRICS prompt was derived from the official web-based form and then iteratively refined using a small set of CC BY–licensed studies, with successive adjustments made at each iteration to progressively improve the accuracy of the results.

Final prompt for analysis

"METRICS Tool v1.0

Please fill out all conditions first for relevant sections and then all active items to calculate METRICS score.

Please note that default option is "No".

? Stands for explanation of items and conditions.

C Stands for conditional items or sections.

Items/Conditions Definitions Weights Options

Study Design

Item#1 ? Adherence to radiomics and/or machine learning-specific checklists or guidelines 0.0368 Yes No

Whether any guideline or checklist, e.g., CLEAR checklist, is used in designing and reporting, as appropriate for the study design (e.g., handcrafted radiomics or deep learning pipeline). Evaluate if the study explicitly references radiomics or machine learning-specific guidelines or checklists (such as CLEAR, CLAIM, or RQS) and mark it as 'Yes' only if explicitly written.

Item#2 ? Eligibility criteria that describe a representative study population 0.0735 Yes No

Whether inclusion and exclusion criteria are explicitly defined. Even when utilizing public datasets, it is essential to assess and clearly define the inclusion and exclusion criteria of that dataset.

Item#3 ? High-quality reference standard with a clear definition 0.0919 Yes No

Whether the reference standard or outcome measure is representative of the current clinical practice and robust. Examples of high-quality reference standards are preferably histopathology, well-established clinical and genomic markers, the latest version of the prognostic tools, guideline-based follow-up or consensus-based expert opinions. Examples of poor quality reference standards are those based on qualitative image evaluation, images that are later used for feature extraction, or outdated versions of prognostic tools."

Imaging Data

Item#4 ? Multi-center 0.0438 Yes No

Whether more than one institution is involved as a diagnostic imaging data source for radiomics analysis. If two hospitals belong to the same institution, they are considered a single center. Additionally, when using public datasets, it is important to verify on the dataset's website whether it includes data from multiple centers.

Item#5 ? Clinical translatability of the imaging data source for radiomics analysis 0.0292 Yes No

Whether the source of the radiomics data is an imaging technique that reflects established standardization approaches, such as acquisition protocol guidelines (e.g., PI-RADS specifications).

Item#6 ? Imaging protocol with acquisition parameters 0.0438 Yes No

Whether the image acquisition protocol is clearly reported to ensure the replicability of the method.

Item#7 ? The interval between imaging used and reference standard 0.0292 Yes No

Whether the time interval between the diagnostic imaging exams (used as an input for the radiomics analysis) and the outcome measure/reference standard acquisition is appropriate to validate the presence or absence of target conditions of the radiomics analysis at the moment of the diagnostic imaging exams.

Segmentation

Condition#1 ? Does the study include segmentation? Yes No

Segmentation refers to i) Fine delineation of a region or volume of interest; ii) Rough delineation with bounding boxes; or, iii) cropping the image around a region of interest.

Condition#2 ? Does the study include fully automated segmentation? Yes No

Fully automated segmentation" refers to segmentation process without any human intervention. If a segmentation technique that does require any sort of human intervention, mark this item as 'No.'.

Item#8 ? Transparent description of segmentation methodology 0.0337 Yes No

Whether the rules or the method of the segmentation are defined (e.g., margin shrinkage, peri-tumoral sampling, details of segmentation regardless of whether manual, semi-automated or automated methods are used). In the case of DL-based radiomics, the segmentation can refer to the rough delineation with bounding boxes or cropping the image around a region of interest.

Item#9 ? Formal evaluation of fully automated segmentation C 0.0225 Yes No

If a segmentation technique that does not require any sort of human intervention is used, examples of the results should be presented and a formal assessment of its accuracy compared to domain expert annotations included in the study (e.g., DICE score or Jaccard index compared with a radiologist's semantic annotation). Any intervention to the annotation in terms of volume or area should be considered as the use of a semi-automated segmentation technique. This item also applies to the use of segmentation models previously validated on other datasets.

Item#10 ? Test set segmentation masks produced by a single reader or automated tool 0.0112 Yes No

Whether final segmentation in the test set is produced by a single reader (manually or with a semi-automated tool) or an entirely automated tool, to better reflect clinical practice. If part of the dataset is segmented by another reader solely for ICC feature calculation, it is still considered as having been segmented by a single reader. However, if the entire dataset is segmented by two readers, it cannot be considered as having been done by a sole reader.

Image Processing and Feature Extraction

Condition#3 ? Does the study include hand-crafted feature extraction? Yes No

Item#11 ? Appropriate use of image preprocessing techniques with transparent description 0.0622 Yes No

Whether preprocessing of the images is appropriately performed considering the imaging modality (e.g., gray level normalization for MRI, image registration in case of multiple contrasts or modalities) and feature extraction techniques (i.e., 2D or 3D) that are used. For instance, in the case of large slice thickness (e.g., ≥5 mm), extreme upsampling (e.g., 1 x 1 x 1 mm3) of the volume might be inappropriate. In such a case, 2D feature extraction could be preferable, ensuring in-plane isotropy of the pixels. On the other hand, achieving isotropic voxel values should be targeted in 3D feature extraction, to allow for texture feature rotational invariance. Also, whether gray level discretization parameters (bin width, along with resulting gray level range, or bin count) are described in full detail. Description of different image types used (e.g., original, filtered) should also be included (e.g., high and low pass filter combinations for wavelet decomposition, sigma values for Laplacian of Gaussian edge enhancement filtering). If the image window is fixed, it should be clarified. In handcrafted feature extraction and machine learning scenarios, at least two of the following four preprocessing steps should be included: image intensity/density normalization, filtering, resampling, and discretization. For deep learning applications, image intensity/density normalization or filtering alone is generally sufficient.

Item#12 ? Use of standardized feature extraction software C 0.0311 Yes No

Whether a standardized software (e.g., compliant with IBSI) was used for feature extraction, including information on the version number. Pyradiomics, ITK-SNAP, CaPTk, RaCaT, and LIFEx are acceptable software options; if any other software is used, please first verify that it is IBSI compliant.

Item#13 ? Transparent reporting of feature extraction parameters, otherwise providing a default configuration statement 0.0415 Yes No

Whether feature types (e.g., hand-crafted, deep features) and feature classes (for hand-crafted) are described. In the case of DL, neural network architecture along with all image operations should be described.

Feature Processing

Condition#4 ? Does the study include tabular data? Yes No

Tabular data refers to data that is organized in a table with rows and columns.

Condition#5 ? Does the study include end-to-end deep learning? Yes No

End-to-end deep learning refers to the use of deep learning to directly process the image data and produce a classification or regression model.

Item#14 ? Removal of non-robust features 0.0200 Yes No

Assess if non-robust features are removed by verifying reproducibility through ICC or similar stability analysis. If ICC or stability analysis is not conducted, mark this item as 'No.'.

Item#15 ? Removal of redundant features 0.0200 Yes No

Whether dimensionality is reduced by selecting the more informative features such as with algorithm-based feature selection (e.g., LASSO coefficients, Random Forest feature importance), univariate correlation, collinearity, or variance analysis.

Item#16 ? Appropriateness of dimensionality compared to data size C 0.0300 Yes No

Please check the AUCs between the training and test sets to ensure similarity. If this consistency is not achieved, it may be beneficial to reduce the total number of features to at least one-tenth of the total labeled data.

Item#17 ? Robustness assessment of end-to-end deep learning pipelines 0.0200 Yes No

Whether DL pipeline consistency of performance has been assessed in a test-retest setting, for example by a scan-rescan approach, use of segmentations by different readers, or stability analysis [i.e., image perturbations]. The specific methods used should be clearly presented.

Preparation for Modeling

Item#18 ? Proper data partitioning process 0.0599 Yes No

Whether the training-validation-test data split is done at the very beginning of the analysis pipeline, prior to any processing step.

Item#19 ? Handling of confounding factors 0.0300 Yes No

Whether potential confounding factors were analyzed, identified if present, and removed if necessary (e.g., if it has a strong influence on generalizability). These may include different distributions of patient characteristics (e.g., gender, lesion stage or grade) across sites or scanners. Additionally, a statistical comparison must be provided in the methods or results section regarding demographic, clinical, and blood test factors that are outside the primary research question to ensure these potential confounders are adequately addressed. Tables presenting these comparisons should also be thoroughly checked and verified for accuracy.

Metrics and Comparison

Item#20 ? Use of appropriate performance evaluation metrics for task 0.0352 Yes No

Whether appropriate accuracy metrics are reported, such as AUC for Receiver Operating Characteristics (ROC) or Precision-Recall (PRC) curves and confusion matrix-derived accuracy metrics (e.g., specificity, sensitivity, precision, F1 score) for classification tasks; MSE, RMSE, and MAE for regression tasks. For classification tasks, the confusion matrix should always be included, to allow the calculation of additional metrics. If training a DL network, loss curves should be presented.

Item#21 ? Consideration of uncertainty 0.0234 Yes No

Whether uncertainty measures are included in the analysis, such as 95% confidence interval (CI), standard deviation (SD), or standard error (SE). Report on methodology to derive that distribution (ie. bootstrapping with replacement, etc). These uncertainty measures should be directly related to the performance metrics of the machine or deep learning models.

Item#22 ? Calibration assessment 0.0176 Yes No

Whether the final model's calibration is assessed. This assessment can include calibration curves and/or Brier scores to evaluate the model's calibration quality accurately.

Item#23 ? Use of uni-parametric imaging or proof of its inferiority 0.0117 Yes No

Use of a single imaging set (such as a single MRI sequence rather than multiple, or a single phase in a dynamic contrast-enhanced scan) should be preferred, as multi-parametric imaging may unnecessarily increase data dimensionality and risk of overfitting. Therefore, in the case of multi-parametric studies, uni-parametric evaluations should also be performed to justify the need for a multi-parametric approach by formally comparing their performance (e.g., DeLong's or McNemar's tests). This item is also intended to reward studies that experimentally justify the use of more complex models compared to simpler alternatives, in regard to input data type.

Item#24 ? Comparison with a non-radiomic approach or proof of added clinical value 0.0293 Yes No

Whether a non-radiomic method that is representative of the clinical practice is included in the analysis for comparison purposes. Non-radiomic methods might include semantic features, RADS or RECIST scoring, and simple volume or size evaluations. If no non-radiomics method is available, proof of improved diagnostic accuracy (e.g., improved performance of a radiologist assisted by the model's output) or patient outcome (e.g., decision analysis, overall survival) should be provided. In any case, the comparison should be done with an appropriate statistical method to evaluate the added practical and clinical value of the model (e.g., DeLong's test for AUC comparison, decision curve analysis for net benefit comparison, Net Reclassification Index). Furthermore, in case of multiple comparisons, multiple testing correction methods (e.g., Bonferroni) should be considered in order to reduce the false discovery rate provided that the statistical comparison is done with a frequentist approach (rather than Bayesian).

Item#25 ? Comparison with simple or classical statistical models 0.0176 Yes No

Whether a comparison with a simple baseline reference model (such as a Zero Rules/No Information Rate classifier) was performed. Use of machine learning methods should be justified by proof of increased performance. In any case, the comparison should be done with an appropriate statistical method (e.g., DeLong's test for AUC comparison, Net Reclassification Index). Furthermore, in case of multiple comparisons, multiple testing correction methods (e.g., Bonferroni, Benjamini–Hochberg, or Tukey) should be considered in order to reduce the false discovery rate provided that the statistical comparison is done with a frequentist approach (rather than Bayesian).

Item#26 ? Internal testing 0.0375 Yes No

Whether the model is tested on an independent data set that is sampled from the same source as the training and/or validation sets. Internal cross-validation methods other than hold-out are not accepted as a positive approach for this purpose.

Item#27 ? External testing 0.0749 Yes No

Whether the model is tested with independent data from other institution(s). This also applies to the studies validating the previously published models trained at another institution.

Item#28 ? Data availability 0.0075 Yes No

Whether any imaging, segmentation, clinical, or radiomics analysis data is shared with the public. Upon request is not accepted for this item.

Item#29 ? Code availability 0.0075 Yes No

Whether all scripts related to automatic segmentation and/or modeling are shared with the public. These should include clear instructions for their implementation (e.g., accompanying documentation, tutorials). Upon request is not accepted for this item.

Item#30 ? Model availability 0.0075 Yes No

Whether the final model is shared in the form of a raw model file or as a ready-to-use system. If automated segmentation was employed, the corresponding trained model should also be made available to allow replication. These should include clear instructions for their usage (e.g., accompanying documentation, tutorials). Upon request is not accepted for this item."

RQS prompt

The RQS prompt was originally developed by Mese I and Kocak B [20] through iterative testing and refinement, including evaluation of model explanations and targeted adjustments to reduce ambiguous scoring and carry-over effects. In the present study, we used the same RQS prompt, except for the "Validation" item, where the score of 0 was replaced with −5 to match the original RQS scoring scheme by Lambin et al [11].

Final prompt for analysis

"I am uploading a scientific paper for analysis. Please assess the paper based on the Radiomics Quality Score criteria provided below. For each criterion, determine whether the paper meets the specified standards and provide a brief justification for your assessment. Here are the criteria and corresponding points:

Image protocol quality: Determine if the image protocols are well-documented (for example, contrast, slice thickness, energy, etc.) and/or if public image protocols are used to allow reproducibility/replicability.

Points: +1 if protocols are well-documented / another+1 if public protocol is used

Multiple segmentations: Check if segmentation is performed by different physicians / algorithms / software / radiologist / neuroradiologist to analyze feature robustness to segmentation variabilities.

Points: +1 if multiple segmentations per instance are carried out. Segmentation verification by another does not counts as multiple segmentations.

Phantom study on all scanners: Verify if inter-scanner differences and vendor-dependent features are detected, and if feature robustness to these sources of variability is analyzed.

Points: +1 if phantom study is used on all scanners

Imaging at multiple time points: Assess if images of individuals are collected at additional time points to analyze feature robustness to temporal variabilities (for example, organ movement, organ expansion/shrinkage).
Points: +1 if images are collected at additional time points. Both images must be used for the radiomics

Feature reduction or adjustment for multiple testing: Determine if measures are implemented to decrease the risk of overfitting. Consider if feature robustness is accounted for when selecting features.

Points: -3 if neither measure is implemented / +3 if either measure is implemented

Multivariable analysis with non-radiomics features: Evaluate if a multivariable analysis including non-radiomics features (for example, EGFR mutation) is performed to provide a more holistic model and to permit correlating/inferencing between radiomics and non-radiomics features.

Points: +1 if multivariable analysis with non-radiomics features is carried out

Detect and discuss biological correlates: Check if the study demonstrates phenotypic differences possibly associated with underlying gene–protein expression patterns, deepening the understanding of radiomics and biology.
Points: +1 if phenotypic differences are demonstrated

Cut-off analyses: Verify if risk groups are determined by either the median, a previously published cut-off, or if a continuous risk variable is reported to reduce the risk of overly optimistic results.

Points: +1 if risk groups are determined by either the median, a previously published cut-off or if a continuous risk variable is reported

Discrimination statistics: Check if discrimination statistics (for example, C-statistic, ROC curve, AUC) and their statistical significance (for example, p-values, confidence intervals) are reported, and if a resampling method (for example, bootstrapping, cross-validation) is applied.

Points: +1 if a discrimination statistic and its statistical significance is reported / another+1 if a resampling method technique is also applied

Calibration statistics: Determine if calibration statistics (for example, Calibration-in-the-large/slope, calibration plots) and their statistical significance (for example, P-values, confidence intervals) are reported, and if a resampling method (for example, bootstrapping, cross-validation) is applied.

Points: +1 if a calibration statistic and its statistical significance is reported / +1 if a resampling method technique is applied

Prospective study registered in a trial database: Check if the study is registered in a trial database, providing evidence supporting the clinical validity and usefulness of the radiomics biomarker.

Points: +7 for prospective validation of a radiomics signature in an appropriate trial

Validation: Evaluate the validation process, determining if it is performed without retraining and without adaptation of the cut-off value, and check if validation provides credible clinical performance.

Points: +2 if validation is based on a dataset from the same institute / +3 if validation is based on a dataset from another institute / +4 if validation is based on two datasets from two distinct institutes / +4 if study validates a previously published signature / +5 if validation is based on three or more datasets from distinct institutes -5 if it did not separate test set from validation/training set.

Comparison method: Assess if the study compares radiomics model to the model that can show the added value of radiomics.

Points: +2 if comparison to the method than can used in clinical routines in most instutions as a reference.

Potential clinical utility: Determine if the study reports on the current and potential application of the model in a clinical setting.

Points: +2 if a test made such as decision curve analysis. +0 if it is only mentioned in the discussion.

Cost-effectiveness analysis: Check if the study reports on the cost-effectiveness of the clinical application (for example, QALYs generated).

Points: +1 if the cost-effectiveness of the clinical application is reported

Open science and data: Verify if the study makes code and data publicly available to facilitate knowledge transfer and reproducibility.

Points: +1 if scans are open source / another +1 if region of interest (ROI) segmentations are open source / another+1 if code is open source / another+1 if representative segmentations and features are open source. +0 if it will be given upon request any of the above-not present within the study or in supplement or in direct website etc.- .”

Distribution of METRICS and RQS points across the four readers (Main Reader, Secondary Readers A and B, and Chat GPT 5.1 Thinking) and items

Supplementary Figure 1 illustrates inter-rater differences across METRICS items. Item-level disagreement between GPT-5.1 and human readers was most pronounced for “Comparison with simple or classical statistical models” (13.3% vs 50.0–56.7%), “Interval between imaging and the reference standard” (76.7% vs 96.7–100%), and “removal of non-robust features” (36.7% vs 50.0–66.7%). Conversely, GPT-5.1 more frequently credited “transparent reporting of feature extraction parameters” (90.0% vs 40.0–66.7%), “test set segmentation masks produced by a single reader or automated tool” (86.7% vs 33.3–66.7%), and “transparent description of the segmentation methodology” (100% vs 66.7–83.3%).

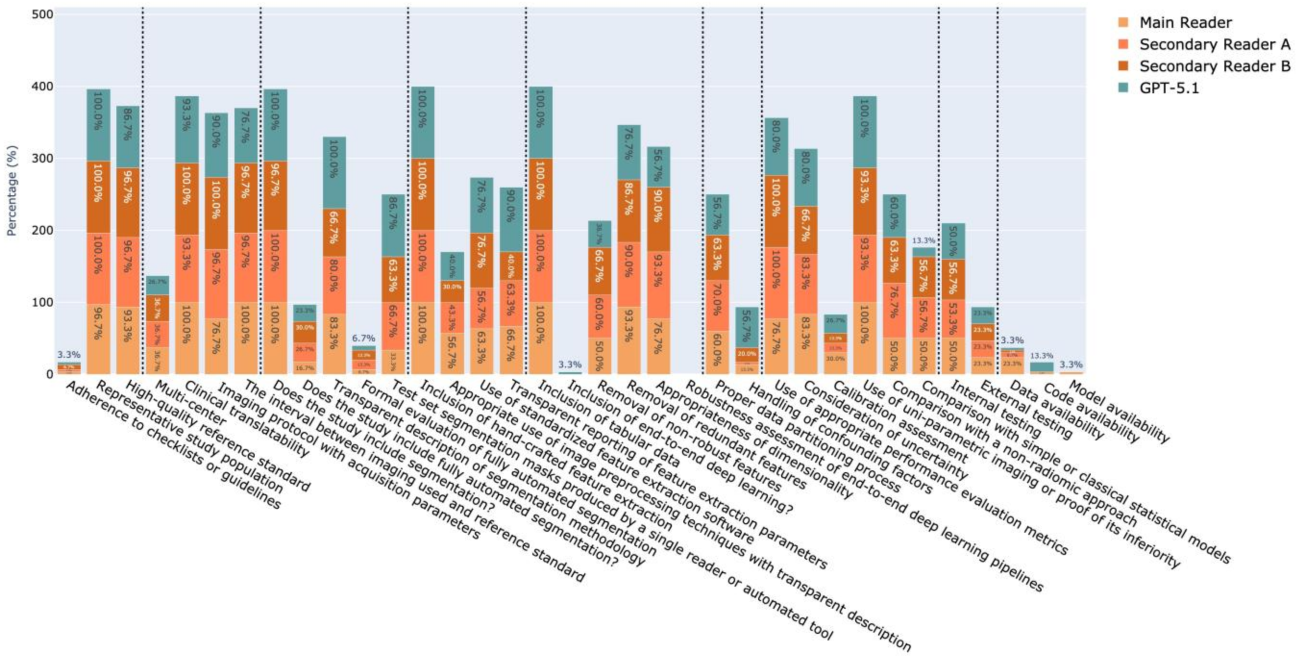

Fig. S1 Proportion of METRICS items scored as positive by the readers (Main Reader, Secondary Readers A and B, ChatGPT 5.1 Thinking) on 30 randomly selected studies. *METRICS* METhodological RadiomICs Score.

Supplementary Figure 2 shows inter-rater differences across RQS items. Item-level disagreement on the RQS between GPT-5.1 and human readers was most pronounced for "clinical utility", where GPT-5.1 assigned the 2-point category in only 2 studies, compared with 28–30 by human readers. Marked discrepancies were also observed for "biological correlates" (0 points: 25 studies vs 6–13; 1 point: 5 vs 17–24) and for "comparison to gold standard" (0 points: 11 vs 1–2; 2 points: 19 vs 28–29). Further differences were evident for "cut-off analyses" (0 points: 19 vs 7–14; 1 point: 11 vs 16–23) and "discrimination statistics" (2 points: 13 vs 4–10; 1 point: 12 vs 16–25; 0 points: 5 vs 0–4).

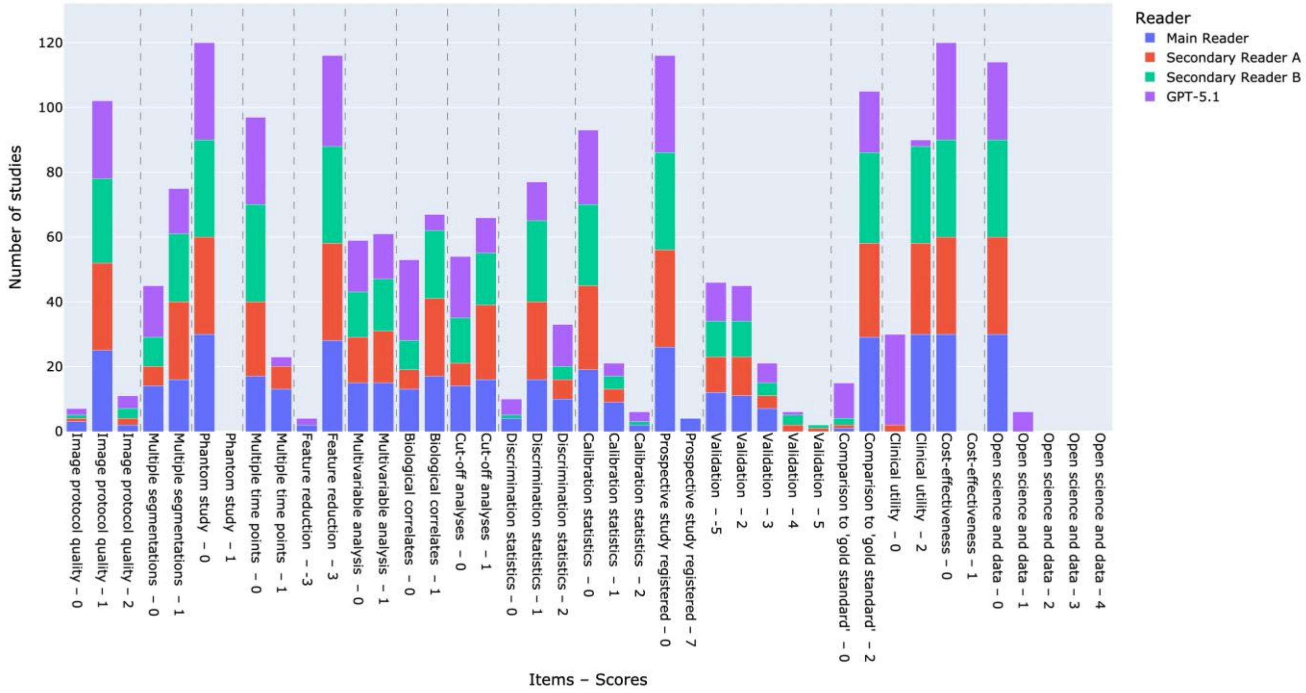

Fig. S2 Distribution of the number of studies across the different RQS items and score levels, according to the readers (Main Reader, Secondary Readers A and B, ChatGPT 5.1 Thinking) on 30 randomly selected studies. *RQS* Radiomics Quality Score.
